# “I didn’t know you could do that”: A Pilot Assessment of EHR Optimization Training

**DOI:** 10.1055/s-0041-1731005

**Published:** 2021-06-27

**Authors:** Rachel Gold, Arwen Bunce, James V. Davis, Joan C. Nelson, Stuart Cowburn, Jee Oakley, Stacie Carney, Michael A. Horberg, James W. Dearing, Gerardo Melgar, Joanna E. Bulkley, Janet Seabrook, Heath Cloutier

**Affiliations:** 1 Kaiser Permanente Center for Health Research, Portland, Oregon, United States; 2 OCHIN, Inc., Portland, Oregon, United States; 3 Department of Primary Care, Kaiser Permanente Northwest, Portland, Oregon, United States; 4 Kaiser Permanente Mid-Atlantic Permanente Research Institute, Rockville, Maryland, United States; 5 Michigan State University, East Lansing, Michigan, United States; 6 Cowlitz Family Health Center, Longview, Washington, United States; 7 Community HealthNet Health Centers, Gary, Indiana, United States

**Keywords:** electronic health records, community health centers, safety-net providers, education, professional, retraining

## Abstract

**Background:**

Informatics tools within electronic health records (EHRs)—for example, data rosters and clinical reminders—can help disseminate care guidelines into clinical practice. Such tools’ adoption varies widely, however, possibly because many primary care providers receive minimal training in even basic EHR functions.

**Objectives:**

This mixed-methods evaluation of a pilot training program sought to identify factors to consider when providing EHR use optimization training in community health centers (CHCs) as a step toward supporting CHC providers’ adoption of EHR tools.

**Methods:**

In spring 2018, we offered 10 CHCs a 2-day, 16-hour training in EHR use optimization, provided by clinician trainers, and customized to each CHC’s needs. We surveyed trainees pre- and immediately post-training and again 3 months later. We conducted post-training interviews with selected clinic staff, and conducted a focus group with the trainers, to assess satisfaction with the training, and perceptions of how it impacted subsequent EHR use.

**Results:**

Six CHCs accepted and received the training; 122 clinic staff members registered to attend, and most who completed the post-training survey reported high satisfaction. Three months post-training, 80% of survey respondents said the training had changed their daily EHR use somewhat or significantly.

**Conclusion:**

Factors to consider when planning EHR use optimization training in CHCs include: CHCs may face barriers to taking part in such training; it may be necessary to customize training to a given clinic’s needs and to different trainees’ clinic roles; identifying trainees’ skill level a priori would help but is challenging; in-person training may be preferable; and inclusion of a practice coach may be helpful. Additional research is needed to identify how to provide such training most effectively.

## Background and Significance

Disseminating evidence-based guidelines into widespread practice is critical to ensuring that patients receive care based on up-to-date medical recommendations. Such dissemination can be expedited when informatics tools such as data rosters and clinical reminders are built into electronic health records (EHRs) and widely used by clinic staff.^1,2^ However, the adoption of such tools—and the approaches used to support this adoption—vary widely.^3–7^ For example, in an academic medical institution, a risk calculator was used in just 64% of eligible encounters, and an order shortcut tool was opened in just 55% of encounters.^5^ In a set of public clinics, providers marked the problem list as reviewed in < 10% of encounters, accepted best practice alerts in <20% of encounters, and printed an after-visit summary in <60% of encounters.^8^ As such tools are designed to enhance care quality and outcomes, identifying effective strategies for increasing their adoption could improve health outcomes in diverse care settings. The need for such strategies is especially pressing in community health centers (CHCs)—clinics that serve our nation’s most vulnerable patient populations.

To identify effective practices for enhancing adoption of EHR-based tools, we conducted a clinical trial (trial registration: ClinicalTrials.gov, NCT02325531) comparing strategies for helping CHC staff adopt a suite of such tools targeting guideline-concordant cardioprotective care, called the CVD Bundle. (These tools were part of an Epic^©^ ambulatory EHR.) In that trial, called the Study of Practices Enabling Implementation and Adaptation in the Safety Net (SPREAD-NET), the main analyses showed that cardioprotective prescribing improved only marginally in most study clinics, and clinics receiving higher levels of support did not improve more than clinics receiving less support (see Refs. ^9^ and ^10^ for details).

To better understand these unexpected findings, we carefully reviewed the interviews conducted with study clinic staff during the intervention period. We found that many clinic staff expressed a lack of comfort and facility using some EHR functions. Several stated a desire for training in using the EHR more efficiently and effectively (referred to here as EHR use optimization training) and some suggested that a lack of general EHR skills may have impacted their ability to implement the CVD Bundle.

This preliminary assessment indicated that while the implementation support strategies tested in our trial focused on adoption of specific EHR tools, some staff lacked a general foundation in EHR use. This likely impacted tool adoption; for example, the CVD Bundle’s panel management tools required a general competence with roster tools, which few clinic staff possessed. In many cases, the 1-day initial orientation on basic EHR functions that clinic staff had received in the past (range = 2–12 years ago) was insufficient to support robust integration of additional EHR features; we also learned that although resources for self-directed learning were available, opportunities for using these resources varied depending on clinic resources. Thus, we surmised that training to optimize general EHR use skills might be needed as a foundation before adoption of specific EHR tools could be effectively supported.

Research is emerging on the impact of providing EHR use optimization training subsequent to initial EHR rollouts in U.S. primary care settings. Several studies in large, integrated care delivery systems found that optimization training improved providers’ EHR skills.^11–14^ A survey of > 72,000 medical staff in diverse provider organizations found that EHR training was associated with improved clinician satisfaction and self-reported care quality.^15^ One study found significant improvements in EHR use proficiency when medical residents received intensive training;^16^ two others, at academic medical centers, found high clinician satisfaction associated with such training.^6,7^

While EHR use optimization training might incur similar benefits in CHCs seeking to enhance adoption of any EHR tools, research on conducting such training in this care setting remains nascent.^17,18^ To begin addressing this knowledge gap, this article describes how we provided one such training and how it was received. Our goal was to identify the feasibility of providing training in EHR use optimization in the CHC setting, and factors that others might consider when planning such trainings.

## Objectives

The objectives of this mixed-methods evaluation were to explore trainee- and trainer-identified factors to be considered when providing EHR use optimization training in CHCs. Our focus was on high-level assessments of what did or did not work well when providing general EHR training as a prelude to training clinic staff about specific EHR tools.

## Methods

This pilot study was designed to identify factors for consideration when providing EHR use optimization training in CHCs. OCHIN, Inc., a nonprofit organization based in Portland, Oregon, United States, provides a shared Epic^©^ EHR to CHC clinics nationwide (at study start, > 400 clinics; at time of writing, > 600 clinics). Clinic selection and recruitment and study clinic characteristics for the SPREAD-NET study are described in previous papers.^9,10^ The 29 CHC clinics in the parent study were managed by 12 organizations; during the study period, one organization with two clinics closed, leaving 27 study clinics. OCHIN’s training team, which provided rollout training and online resources for the parent study described above, also provided the training described here.

In spring 2018, the study CHCs were offered EHR use optimization training involving 2-day visits to each organization, up to 16 hours of training. Two training designs were offered: 2 hours of individualized training per trainee for ≤ 8 providers, or 4 to 8 hours of classroom-style training for ≤ 4 groups of ≤ 12 trainees. The trainings offered would cover general EHR use optimization, with training content customized to each clinic’s needs as described below, as long as the CVD Bundle was covered. Staff such as physicians, nurses, medical assistants, and the clinics’ EHR specialists were invited to attend; participating clinics were allowed to include staff as desired, with no formal recruitment of trainees or other inclusion criteria. Attendance at the training was optional for clinic staff at some sites and required by clinic leadership at others.

A 4-phase training plan was developed in which training content and format could be adapted to meet the needs of each clinic and the trainers’ preferences.

- Phase 1: Assess needs. Ten weeks prior to the training, a clinic representative completed a questionnaire asking which content areas should be covered (Table 1). The trainers also used Epic^©^ software that quantifies EHR use patterns to assess each organization’s usage for areas of inefficiency. The trainers discussed these findings with each organization, as they finalized their training plan.
- Phase 2: Onsite Day 1. The trainers spent 2 to 4 hours observing EHR use, then discussed EHR use practices with trainees. The trainers then recommended aspects of needed optimization training based on these observations and on information from Phase 1. The trainers then customized a list of optimization-related topics to cover at the training and reviewed the list with clinic leadership.
- Phase 3: Onsite Day 2. Clinic leaders (e.g., Chief Medical Officer, Chief Operations Officer, EHR specialists) introduced the trainers so the trainees would know that the training was a clinic priority. The trainers then delivered the training via the clinic’s chosen modality (classroom or one-on-one), including demonstrating EHR functionalities that the trainees then practiced.
- Phase 4 (post-training): Where appropriate, trainees were emailed links to resources on training content areas, including user guides and videos on using specific EHR tools.

The trainings were conducted in late spring 2018. Two of seven trainers went to each trainee site, with one exception involving only one trainer. All of the trainers were clinicians (Table 2). Trainers spent 8 to 16 hours per site over 2 days. Trainees varied by site, ranging from: 8 providers and support staff; 24 providers and support staff (across two sessions); one-on-one training for 8 providers; 6 support staff; and 40 providers (across four sessions). One organization opted for the one-on-one training only; the rest included some group training.

### Data Collection and Analysis

#### Surveys:

Trainees were surveyed in person pre- and immediately post-training, and again 3 months later via email (Tables 3–5). The surveys, developed by OCHIN’s training team, included measures commonly used in assessments of other trainings provided to OCHIN member CHCs. They asked about satisfaction with the training, which aspects of the training were valuable and which could be improved, and how the training was expected to impact or had impacted trainees’ subsequent EHR use. Specific survey questions are shown in Tables 3–5. Survey results were treated as descriptive data; no statistical analyses were conducted.

#### Process data:

We documented: number of organizations accepting the training offer; number of clinic providers and other staff who attended the trainings; training method (1:1 vs. group); requested/provided training content; and time spent on the training.

#### Qualitative data:

The parent study included a qualitative process evaluation designed to identify routine implementation challenges, to better understand intervention outcomes. It is summarized here and described in detail in prior publications.^9,10^ In brief, over 3 years, researchers regularly (biweekly, then monthly, then quarterly) called the staff member assigned to lead implementation activities at each CHC to ask about such barriers. At these calls, the clinic contacts were asked about implementation activities, challenges, solutions, and surprises, as well as staff reactions to and use of the targeted EHR tools and implementation support.

After the trainings described here, a question was added to these check-ins to ascertain trainee reactions to the trainings; the subject also occasionally arose organically during these conversations.

We also conducted a post-training focus group with all seven trainers. The trainers were asked for their thoughts on: the effectiveness of different training approaches (e.g., training separated by staff role vs. not; over-the-shoulder vs. classroom style; standardized vs. flexible curriculum); any modifications that were made, or suggested for the future; factors that may have impacted trainee engagement (e.g., background of trainer; involvement of clinic leadership); and their perception of the impact of the training in general. The focus group and all interviews were recorded and transcribed verbatim.

Qualitative analyses in the parent study were guided by the constant comparative method^19^ and conducted in QSR NVivo; code development followed a formal team-based process in which codes and code definitions were identified and iteratively refined; 5% of the transcripts were double coded to ensure coding consistency.^9^ As the work discussed here occurred at the end of the parent study, we created a single new code to capture all data related to the optimization trainings. Data for this analysis drew from the trainer focus group, the check-in calls with clinic contacts, and email communications between trainers and clinics. During analyses we reviewed the coded data to identify themes and variations across clinics and participants. Findings from the surveys and the qualitative data were highly congruent. We selected quotes that were representative of the overall findings.

## Results

Six of the 10 CHC organizations (60%) accepted the offered EHR use optimization training. Two of the four organizations that declined provided reasons for not participating. One organization noted concerns that the training would emphasize EHR use patterns that differed from the clinic’s established workflows; the effort involved in planning for it; the reluctance to reduce access to care due to staff time spent at the training; and the financial impact of taking providers away from patient care. The second organization noted that the training was inconvenient because clinic staff had too many competing demands on their time.

The six organizations opting for the training oversaw 13 study clinic sites located in 4 states (OR, MT, CA, OH); one was a Rural Health Center and the rest were Federally Qualified Health Centers in urban areas. All had been on their current EHR system for at least 5 years at the time of the training. They varied in size from 2 to 10 full-time equivalent primary care providers and between 1,200 and 6,893 patients annually, with patient populations ranging from 8 to 96% white, 2 to 87% Hispanic, 3 to 19% uninsured, 1 to 47% privately insured, and 35 to 89% publicly insured.

Contacts at these CHCs requested that the training format be informal (e.g., minimally didactic); let trainees apply what they were learning at the training in real time; be led by clinicians; and involve some hands-on support. Table 1 shows the training content and format chosen by the trainee organizations in Phase 1. When asked to identify specific training needs, all six organizations selected “General EHR Optimization.” Five (83%) selected “Support staff training.” Four (67%) selected “User-specific screen optimization” and “Using dotphrases” (text shortcuts). Three (50%) selected “Using Synopsis / Snapshot (patient data summaries)”; two (33%) selected “Reporting Workbench” (panel management / roster tool); one (17%) selected “Personalization Labs” (EHR customization for a given user); and none selected “Learning after go-live” (see Table 1).

### Trainee Perspectives

One hundred twenty-two providers and clinic staff registered to attend training. Of the registered trainees, 49 (40%) completed the pre-training survey, 41 (34%) completed the immediate post-training survey, and 30 (25%) completed the 3-month post-training survey. The majority of respondents who completed the pre-training survey were clinicians (86%).

Tables 3–5 show results of the pre- and post-training surveys. Most pre-training survey respondents (74%) said they use the EHR “pretty well.” The majority of respondents who completed the immediate post-training survey (76%) reported being very satisfied with the training and said it would change their daily EHR use significantly (34%) or somewhat (51%). Of the 30 trainees who completed the 3-month post-training survey (Table 5), most (80%) said the training changed their daily EHR use somewhat or significantly. The most common take-aways from the training that the trainees reported using were user-specific screen customizations (reported by 40% of respondents), Synopsis (patient data summaries; 13%), and Smart Tools (documentation shortcuts; 17%).

Due to staff attrition, we were only able to speak with six individuals from four [#3, 4, 5, and 6] of the six organizations that participated in this training. Four of these (at four separate CHCs) had themselves participated in the training; the others reported on relevant post-training conversations and actions of staff who took part. Trainees’ post-training perceptions, as reported during interviews, were uniformly positive. They frequently noted that they found the training practical and usable, were surprised by how much they had not known about using the EHR efficiently, and had learned easier ways of navigating the EHR that facilitated tasks they had been doing for years. A provider noted: “At first I thought, oh gosh, you know, there really is a lot within [the EHR] that I’m not familiar with. But I wasn’t the only one. I think more individuals than not were … just unaware of how you could access information about the patient … it was very helpful” [#6]. A medical assistant said: “It was nice seeing everything that you could do … and how easy it is. I was just like shocked … I didn’t know you could do that … I’m playing with it [the EHR dashboard] more than I ever did, because I was kind of intimidated by it and I didn’t want to touch it” [#4].

Respondents also shared examples of specific post-training changes they made in their EHR use, including reconfiguring the patient list, signing patients up for a portal account in the exam room, modifying the EHR dashboard, and generating panel-level data for an upcoming site visit. A pharmacist reported that a provider told her that “[the trainer] showed them how to go back like through the problem list and just see kind of like a running list of the assessment and plan so you didn’t have to go back through all the particular notes” [#5] and that she changed her documentation practices as a result. A medical director noted: “I changed my template after I met with [trainer]. … [Now] people are seeing my new template, so they’re asking me how I did that. And so I think it was helpful because we’re seeing some of the changes and then we’re all kind of wanting to implement them. And so, yeah, I would say it’s … it’s brought up a lot of conversation” [#5]. One provider mentioned her frustration with the cumbersome process of accessing a patient’s CVD risk score during the training, and reported that: “And, you know, then the response was, oh! Just go to, you know, where…where the listing of your patient is and you can reconfigure … your daily list of patients you’re gonna see. And, if it’s appropriate given the age of the patient, your ASCVD risk will pop right in. So I have that for all of mine now, right now.” The same provider also noted, regarding lessons gained from the training: “it’s making my life easier. And more importantly, … it is helping to enhance the care for the patients” [#6].

Many trainees appreciated that the training’s hands-on structure let them try out new ways of using the EHR at their clinic and apply changes as they were demonstrated. A medical director [#5] said she appreciated the opportunity to modify her EHR’s templates during the training, as she would not otherwise have time to do so. Some trainees found it helpful having the trainer suggest potential EHR use improvements in an “elbow-side” manner. An informatics supervisor [#3] noted that the trainers’ clinical backgrounds gave them credibility among provider trainees.

Most of the trainings involved trainees with a mixture of clinic roles. Some felt this enabled cross-role understanding. Others noted the challenge of meeting different trainees’ needs, be it based on difference in roles or EHR experience. For example, “the feedback I got from my pharmacist was … that a lot of it applied more to the providers and less like to her. So I would have loved to have, you know, just had a pharmacy group. Or, you know, maybe an MA group, or a provider group” [#5]. A Clinic Operations Officer at the same clinic thought the training was less beneficial for support staff than it was for providers.

Some noted that taking part in the training was challenging due to time demands and the need to take providers away from patient care (precisely why some sites declined). One appreciated allowing the clinics to drive the content and structure of the training: “So, at first I was like, no. Not interested. And then I…I read through it again. And I was like, oh. They’re saying they’re willing to augment the training to whatever we want, or what we need … Then I was like, oh yeah. Okay. This changes everything” [#3].

### Trainer Perspectives

Seven trainers were involved in these trainings. The trainers thought the trainings were well-received overall, and that the trainees learned new skills. For example: “And so there were a lot of…light bulbs going off, as they were realizing that they could customize things, because they just thought that this was the way it was” [Trainer #7].

The trainers identified challenges related to planning training content, such as not knowing variation in EHR skill level between sites prior to the training. They reflected that the pre-training process in which clinics were asked to identify their staff’s training needs did not adequately capture these needs, perhaps because the staff member completing the prequestionnaire was usually an administrator rather than a trainee, and thus unaware of potentially useful EHR tools or functions. To address this, the trainers suggested asking trainees to demonstrate specific skills beforehand; for example, by asking: “Not just, hey, what do you think would be good to, you know, work on? Or what are you weak on? I think you would have to give more examples like, if I ask you build a SmartPhrase [text shortcut], could you do it? … If I ask you to customize at least three wrenches on your screen, could you do it? … Because they don’t know what they don’t know” [Trainer #3]. Some trainers also noted the challenge of balancing standardization (e.g., using a pre-set curriculum) and flexibility in training content; a suggested compromise was a standardized toolbox that trainers could pull from, while still customizing the training to meet local needs.

The trainers also noted that contextual factors such as clinic structure and workflows might influence the effectiveness of EHR use optimization training. To address this, one trainer suggested that EHR training should include a practice facilitator, because “Two kinds of skills are needed to address the problems, because it’s always computer plus people and workflow” [Trainer #5]. Trainers also remarked on the importance of visible leadership support at the trainings, suggesting that it signaled that clinic leadership prioritized the training, thus encouraging greater attentiveness from staff.

Finally, user perception of automated EHR tools’ accuracy is a known barrier to their adoption.^4,20^ One trainer noted that this was addressed when the training focused on the CVD Bundle’s components: “there was one Medical Director who said this is really helpful to know that it’s so robust. Because otherwise, all you see is [an alert]. And the first question you have is why is the system trying to tell me what to do? Right? And now that you know, oh, actually, clinicians built this. And there’s a lot of work and a lot of logic behind it, now we can trust it more, you know” [Trainer #2].

## Discussion

This article describes trainee and trainer perceptions of a pilot effort to provide general EHR use optimization training as a step toward improving adoption of specific EHR tools in primary care CHCs. It adds to the literature by highlighting trainee and trainer perspectives on such training, and some reported benefits of this training, such as potentially increasing trainees’ EHR skills and adoption of specific EHR tools.

Our findings have implications for research involving EHR-based tools. The SPREAD-NET study’s qualitative findings indicate that clinic staff were asked to adopt such tools without first ensuring that their EHR skills were adequate. This suggests the potential benefits of assessing EHR users’ basic skills prior to testing the adoption and impact of EHR-based interventions.

Future efforts to use EHR use optimization training in CHCs should consider the following points, as indicated by these results:

- CHCs’ barriers to taking part in EHR use optimization training can include: concerns that such training would emphasize EHR use patterns that misalign with existing workflows and so would add confusion if applied; a reluctance to ask more of overstretched providers; the costs of planning for such training; and the cost of participating, in terms of staff time away from patients. All these concerns were noted by clinics that were offered the training.
- Optimization training should be customized to a given clinic’s needs, as trainees at different clinics are likely to have varying EHR skills and different priorities in using the EHR. Yet some standardization in training content is also desirable, to avoid inconsistency and ensure that topics related to the specific EHR tool to be implemented are covered. This tension between customization and standardization was highlighted in the trainers’ observations.
- It may be useful to ensure that trainings explain the rationale for and development of automated EHR tools, including who built them, to support users’ trust in the tools. The benefit of knowing these factors was noted by trainees.
- Identifying trainees’ skill level a priori is challenging given that methods for assessing EHR users’ skills in general—let alone as an indicator of their training needs—have not been validated. Asking trainees to demonstrate specific skills beforehand may help assess proficiencies. The trainers in this study strongly emphasized this point.
- Planners should consider whether to provide training in person versus remotely. While remote training is far less costly, the results presented here emphasize that trainees appreciated the hands-on nature of trainers coming to the clinic.
- Planners should consider whether to provide training to trainees with the same versus different clinic roles, and how to adapt a training to mitigate the downsides associated with these choices.^16^ If a mixed-role training is desired, trainers should consider how to make training content useful to all attendees. As presented above, trainees called out the pros and cons of taking part in trainings whose trainees had diverse staff roles.
- Trainees appreciated having a clinician-trainer—someone who uses the EHR in practice. However, clinicians can be expensive, and clinician informaticists are rare. It might be useful to have a practice facilitator on the training team because optimized use of EHRs is impacted by knowledge of available tools, but also by clinic workflows and structures within which the tools are used—factors that a coach could help address. As noted in the “Results” section, this point was raised by the trainers in recognition of the limitations of the skills they brought to the trainings.

The need for EHR use optimization training in primary care settings, identified previously,^15^ is underscored by these results. Most trainees were surprised by how much they learned about using the EHR efficiently, and about how to use the EHR to easily complete standard tasks; many reported applying EHR use techniques from the training to such tasks. Some trainees said learning these skills helped address fears about trying out new things within their EHR. Given these outcomes—and in light of recent research showing that such mastery improves user satisfaction^15,17^—further evidence is needed on when and how to provide EHR use optimization training in primary care settings. Specifically, research is needed to validate methods for identifying trainees’ EHR skills and training needs, provide hands-on training effectively, and develop self-guided EHR trainings that can provide the benefits of hands-on, in-person training, and to better understand the costs involved in clinics taking part in such trainings. Evidence from research on initial EHR implementation may provide useful guidance on EHR use optimization approaches. For example, McAlearney et al^21^ describe the importance of trainees’ perception that a given behavior change will yield a benefit, gaining self-efficacy, and the context where training occurs, when implementing EHRs; our results show these factors’ importance in trainings targeting improved EHR use.

### Limitations:

This evaluation was not designed to assess the impact of providing EHR use optimization training on EHR use patterns, care quality, or patient outcomes, nor to compare training approaches. The number of participating sites was small, and we were not able to assess impacts on CVD Bundle adoption post-training. Rather, it was designed to provide a preliminary assessment of factors to consider when providing such training, and to generate hypotheses for future research. Data collection to evaluate trainees’ pre- and post-training perceptions was conducted as feasible, but we did not conduct a systematic or exhaustive evaluation.

Our results yield preliminary lessons on factors involved in providing EHR use optimization training (e.g., the benefits of customizing training structures to meet individual clinics’ needs) in CHCs. They are presented here to inform future efforts to conduct such trainings in general or as precursors to adoption of new EHR tools, not to enable direct replication of a specific curriculum. However, high-level learnings from this work could be adopted by others developing such curricula, for example, the benefits of customizing training to meet site needs.

The cost of providing this training was covered by the parent study; efforts to provide EHR use optimization training in the future must identify funding methods, and research on the cost benefits of such training is needed. We did not collect data on the percentage of each clinic’s staff that participated in the training; future research should assess this aspect of training success. The surveys used to assess satisfaction with the trainings were not based on validated measures but rather were designed to align with other trainings provided by that team; future research should use validated measures. Last, it was not feasible to conduct pre- and post-training assessment of EHR use patterns: it was out of scope in this evaluation, and validated measures of such patterns were not available at the time of the study. Future research is needed to assess such impacts.

## Conclusion

EHR use optimization training holds potential for improving clinic staff’s ability to use their EHRs more effectively and efficiently. The training and clinical informatics teams at OCHIN, our study site, building on this work, are developing additional EHR use optimization training services to offer to OCHIN’s member CHCs. These results highlight the need to consider site-specific EHR-related strengths and weaknesses prior to launching a large implementation study, as well as factors to consider when providing such training in CHCs, and points to the need for additional research to identify how to provide such training most effectively.

## Figures and Tables

**Table 1:** Results of pre-training clinic evaluation

Questions		Organization					
		1	2	3	4	5	6
# of staff registered to attend training (*n* = 122)		8	36	40	6	8	24
Preferred modality	Classroom		X	X	X		X
	Shoulder-to-shoulder (one-on-one)	X				X	
Topics of interest for training content	General EHR use optimization	X	X	X	X	X	X
	User-specific screen customization	X			X	X	X
	Personalization labs				X		
	Support staff training	X	X		X	X	X
	Panel management/roster tools	X			X		
	Using synopsis/snapshot			X	X	X	
	Using dotphrases			X	X	X	X

Abbreviation: EHR, electronic health record.

**Table 2:** Trainer characteristics

Trainer	Degrees	Role	Organization					
			1	2	3	4	5	6
1	MSN, FNP, NP-C	Clinical informaticist		X			X	
2	MD	Clinical informaticist			X			X
3	MSW	Clinical informaticist						X
4	MPH, PA-C	Practice coach		X				
5	MD	Clinical informaticist	X			X		
6	RN	EHR trainer	X		X			
7	PT	Clinical programs manager				X		

Abbreviation: EHR, electronic health record.

**Table 3:** Pre-training survey results by organization

	Organization						Total
	1	2	3	4	5	6	
Responses (*n*)	6	2	36	5	0	0	49
Clinic role							
Provider	5	1	36	0	0	0	42
EHR specialist	1	0	0	1	0	0	2
Clinical support staff	0	1	0	2	0	0	3
Other	1	0	0	2	0	0	3
How well do you feel you use the EHR in general?							
Extremely well	0	0	2	0	0	0	2
Pretty well	5	0	26	5	0	0	36
Neutral	1	1	6	0	0	0	8
Not very well	0	1	2	0	0	0	3
What do you hope to gain from this training?							
Increased general EHR proficiency	4	2	26	4	0	0	36
Customize screen to better support individual workflow	2	0	2	2	0	0	6
Better understand how to use specific EHR features	1	0	6	0	0	0	7
Other	0	0	4	0	0	0	4

Abbreviation: EHR, electronic health record.

**Table 4:** Immediate post-training survey results by organization

	Organization						Total
	1	2	3	4	5	6	
Responses (*n*)	3	2	36	0	0	0	41
How would you rate your overall satisfaction with this training?							
5-Very satisfied	3	0	28	0	0	0	31
4-Somewhat satisfied	0	0	8	0	0	0	8
3-Neutral	0	1	0	0	0	0	1
2-Somewhat not satisfied	0	0	0	0	0	0	0
1-Not satisfied	0	1	0	0	0	0	1
How much do you think this training will change your use of the EHR in your daily practice?							
4-Significantly	2	0	12	0	0	0	14
3-Some	1	0	20	0	0	0	21
2-Very little	0	2	4	0	0	0	6
1-Not at all	0	0	0	0	0	0	0

Abbreviation: EHR, electronic health record.

**Table 5:** Three-month post-training survey results by organization

	Organization						Total
	1	2	3	4	5	6	
Responses (*n*)	2	9	10	4	2	3	30
What learnings from the training do you now use regularly?							
User-specific screen customization	2	3	6	0	0	1	12
Reporting workbench	0		0	2	0	0	2
Using synopsis	0	2	1	0	0	1	4
Using snapshot	0	1	1	0	0	0	2
Using SmartTools	0	2	1	1	0	1	5
Other	0	1	1	1	2	0	5
Other responses:	Helping provide additional support to providers Using BPA and shifting workflows to [use] this / other BPAs consistently Templates / shortcuts Able to use Wrap-Up more efficiently						
How much has the training changed your use of the EHR in your daily practice?							
4-Significantly	0	1	2	0	0	1	4
3-Some	2	4	6	4	2	2	20
2-Very little	0	3	2	0	0	0	5
1-Not at all	0	1	0	0	0	0	1

Abbreviation: EHR, electronic health record.

## Clinical Relevance Statement

Uptake of care guidelines into practice can increase when clinic staff use clinical decision support tools that are built into electronic health records (EHRs). However, adoption of such tools is suboptimal, possibly because many primary care staff receive little training in the effective use of EHRs. These results highlight factors to consider when providing EHR use optimization training in community health centers and suggest that providing such training might improve adoption of EHR-based tools designed to improve care quality.
